# Healthcare system development in the Middle East and North Africa region: Challenges, endeavors and prospective opportunities

**DOI:** 10.3389/fpubh.2022.1045739

**Published:** 2022-12-22

**Authors:** Maram Gamal Katoue, Arcadio A. Cerda, Leidy Y. García, Mihajlo Jakovljevic

**Affiliations:** ^1^Department of Pharmacology and Therapeutics, College of Pharmacy, Kuwait University, Kuwait, Kuwait; ^2^Faculty of Economics and Business, University of Talca, Talca, Chile; ^3^Institute of Advanced Manufacturing Technologies, Peter the Great St. Petersburg Polytechnic University, Saint Petersburg, Russia; ^4^Institute of Comparative Economic Studies, Hosei University Faculty of Economics, Tokyo, Japan; ^5^Department of Global Health Economics and Policy, University of Kragujevac, Kragujevac, Serbia

**Keywords:** MENA, health reform, health financing, health system performance, health outcomes, health workforce

## Abstract

**Background:**

Countries in the Middle East and North Africa (MENA) region have been investing in the development of their health systems through implementing reforms to improve health care delivery for their nations. However, these countries are still facing challenges in providing equitable, high quality healthcare services. There is limited published literature supporting the previous and ongoing attempts that have been made to improve health system performance in MENA countries.

**Aims:**

This review aims to describe experiences of health system development efforts in the MENA region, highlight progress, identify challenges that need be addressed and future opportunities to achieve responsive and efficient health systems. It also aimed to provide recommendations to further support these health systems toward evolution and performance improvement.

**Methods:**

A literature review was conducted by searching different databases including PubMed, Scopus, Google Scholar and other electronic resources to identify articles and publications describing health systems development in the MENA region from 1975 to 2022. It also included grey literature, reports and policy and planning documents by international organizations. The identified references were reviewed to extract, analyze, organize and report the findings.

**Results:**

The review revealed emerging evidence describing governmental initiatives to introduce health system reforms at different levels in the MENA countries. These include initiatives targeting the various elements controlling health system reform: financing, payment, organization, regulation and behavior of providers and consumers. There are several challenges facing the health systems of MENA countries including the rising burden of chronic diseases, inequitable access to health services, deficiency in health workforce, shortage in the use of effective health information systems and leadership challenges. The review identified several key areas that can benefit from further improvement to support health system reforms. These include improving the structure, organization and financing of health systems, health workforce development, effective data management and engagement of key stakeholders to achieve adequate health system reforms.

**Conclusion:**

The MENA countries have made significant steps to improve the performance of their health systems; yet achieving a comprehensive health reform will require collaboration of various stakeholders including health policy makers, healthcare professionals, and central to the success of the reform, the patients.

## Introduction

The MENA region includes several countries and territories geographically localized in the Middle East and North Africa (1). There is a wide variation among these countries with respect to demographic trends and dynamics, as well as in Gross National Income (GNI) (1, 2). Most of the MENA countries are at a stage of medium to high population growth and fertility, while some countries are at advanced phase of demographic transition (1). The population is mainly urban and young, with more than half of the population under the age of 25 (1, 3, 4).

More than half of the MENA countries contribute significantly to the energy production of the world (4). Despite the wealth of resources, there has been modest progress and reduction of poverty in the region in comparison with other countries (4). These countries vary considerably in their economic status and own various economic drivers, including oil, tourism, agriculture and manufacturing (3). MENA countries can be classified into three categories according to their economic and health outcomes achievements (4, 5). These includes (1) low-income countries which have the top infant death rates and maternal mortality ratios in the region that are witnessing the most significant challenges in health care (e.g., Yemen and Djibouti); (2) middle-income countries which have achieved substantial improvements in health outcomes even though some of these countries are challenged by rural/urban variations in health outcomes and fragmentation in health coverage (e.g., Algeria, Egypt, West Bank and Gaza, Jordan, Lebanon, Iran, Iraq, Syria, Libya, Morocco and Tunisia); and (3) high-income countries which have accomplished significant health outcomes as a result of oil revenues, e.g., the Gulf Cooperation Council (GCC) countries (Saudi Arabia, Kuwait, Qatar, Bahrain, the United Arab Emirates and Oman) (4, 5).

Over the years, political uprisings, protests, and armed conflicts have affected several countries in the MENA region and some large-scale conflicts still exist in countries such as Syria, and between Gaza and Israel to date (4). In addition, the following issues continue to pose major development challenges to the governments of the MENA countries: rapid population growth, high unemployment rates especially amongst the youth, gender inequality, scarcity of water resources and socioeconomic gaps between the rich and poor (4). Improving health care delivery services is a key to long-term stability of these countries (3).

All countries are striving to afford their population access to health services in the most efficient way through providing universal health coverage (UHC), a concept that has been supported by the United Nations (UN) and World Health Organization (WHO) (6). This implies the access to quality health services for all people without the risk of exposure to poverty as a result of payment for these services (7). This means that all people can utilize effective preventive, promotive, curative, palliative and rehabilitative health services that they need, while ensuring that this will not expose them to financial hardship (8). Primary health care is the basis of UHC (9). However, providing an affordable health care is a challenge facing all countries, as new therapies are developed and resource allocation demands increase (10). Demographic shifts including the increase in the size of the aging population put an enormous pressure on health systems to provide suitable care for the elderly patients who tend to experience co-morbidities, cognitive decline and fragility more than younger patients (10).

The MENA countries have achieved significant developments in their health systems and the health outcomes of their population over the past decades (3, 4, 11, 12). These involved improving health service delivery, developing public health programs and adopting new medical technologies, as well as educational and socioeconomic developments (4). These countries have achieved significant improvement in providing population access to basic health services (13). This has significantly improved morbidity and mortality patterns and other measures of health status including the mean life expectancy at birth (LEB) (3, 4, 11, 14). There has been a significant progress in the reduction of child mortality, improvement in maternal health and management of communicable diseases in MENA countries (3, 14). Indeed, the decline in child mortality in these countries has been achieved at rates faster than other developing countries in the world (13). Yet, there are significant variations among and within countries of the MENA region in these achievements (4, 11).

However, the MENA region still faces a multitude of political, macroeconomic, social and health challenges (3). These include the need to ensure social and political stability, equitable and comprehensive social and economic rights, and to expand the opportunities of employment for its population (1). Social justice in health care aligns with the principles of UHC (7). In spite of the progress achieved in the health systems of the MENA countries, people living in some of these countries still suffer from inequities in some health outcomes including maternal and child health outcomes (13). Examples include people living in the Gaza Strip, Iraq and Yemen who have experienced deterioration in health outcomes due to conflict-related problems (13).

The MENA region includes a wide range of different health systems in countries that share similar linguistic, cultural and historic backgrounds (15). There are significant differences among these countries in key parameters including per capita national income, percentage of total health expenditure (THE) out of gross domestic product (GDP), life expectancy and average years of schooling (15). The health systems in these countries have been increasingly facing multitude of challenges to deliver quality health services to the population (3–5, 13). To overcome the challenges facing the health systems in the MENA region, this requires a strong political commitment to implement rigorous efforts and consistent reforms to meet the population health needs, especially at a time of limited fiscal resources (16).

Health reform involves polices and measures taken by governments to improve health care delivery to the population of a country (17). The aim of this process is to expand healthcare coverage for the population and improve healthcare services through organizing policy-initiated changes that are achieved through operational, financial, systems, process or practice interventions (17). Examples include implementation of new or renewed safety and quality practices, application of advanced technology to support care improvement, achievement of efficiencies in health care delivery, and ensuring the availability of reliable information to facilitate cost-effective and appropriate healthcare decisions (18, 19).

**Figure 1:**
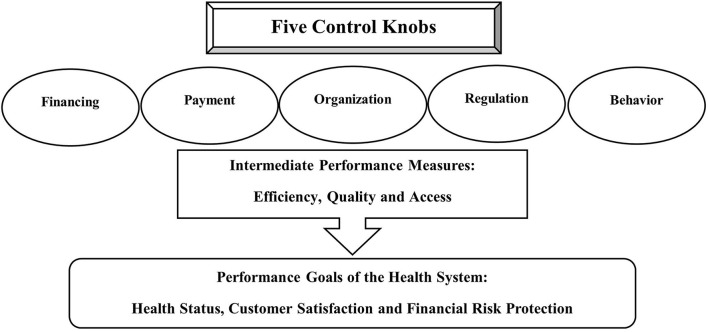
Visual illustration of the elements of the Flagship Framework.

Roberts et al. (17) in their textbook “Getting health reform right: a guide to improving performance and equity” proposed the control knobs framework for assessing health systems and facilitating health reform. This framework (The Flagship Framework) assigns five key mechanisms or processes (knobs) that can be adjusted or changed to design effective health reform and improve health system performance as illustrated in Figure 1 (17). These five knobs include *Financing*, which involves all the resources, including the mechanisms and activities intended to gather funds for the healthcare system such as insurance premiums, health-related taxes and out-of-pocket (OOP) expenses (17). This knob which is determined according to the country's politics and social values has significant impact on the access to health care, protection from financial risk, and the health status of the population and particular groups within the population (17). The second knob is *Payment* which reflects allocation of existing resources to health services providers (17). The third knob is the *Organization* of the health system which describes the structure of the providers of care, their roles, activities and operations, and structure of the care market (17). The forth knob is the *Regulation* which refers to the policies and actions that change the behavior of the different stakeholders in the healthcare system, including the healthcare providers, individual consumers, medical associations, insurance agents and others (17). Lastly, the *Behavior* knob which aims to change individual behavior (of both providers and patients) through population-based interventions to improve the performance and outcomes of the healthcare system (17).

Health reforms can be reflected by improving three intrinsic performance goals of the health system: the health status of the target population, customer satisfaction and financial risk protection (the ability of health system to secure the population from the fiscal burden of poor health or diseases) (17). Moreover, the authors determined three intermediate performance measures that reflect the performance of health system goals: efficiency, access to, and quality of care (16, 17). The Flagship Framework has been increasingly utilized to organize health systems planning, thinking and reform (16).

Reforms are usually complex; their success depends on multifaceted and interlinked factors and can be influenced by many elements, including the country's economy, geography, culture, population size, socioeconomic circumstances and its political structure and stability, along with several other variables (19). Achieving specific outcomes of a health reform might also require the fine-tuning of several knobs concurrently with no specific order of turning the control knobs (17). Health reforms also differ by settings and a successful reform in one context may not necessarily be applicable to another (17). This can be explained by the impact of cultural and structural factors in a given context on the actions of these knobs (17). However, each country can achieve successful health reforms. Evidence has shown that different countries from across the globe were able to report a successful or nearly successful reform experience, irrespective of their political or economic situation (20).

There is limited literature describing the previous and ongoing initiatives for development of health system performance in MENA countries. This review aims to describe examples of health system development efforts in countries of the MENA region and to outline them in light of the Flagship Framework, highlight the progress, identify the challenges facing these countries and the future opportunities to serve the health needs of their population. It also aimed to provide recommendations to further support the success of the efforts for achieving successful health system reforms in the MENA region.

## Methods

A comprehensive literature review was conducted by searching different search engines and databases including PubMed, Scopus, Google Scholar and other electronic resources during the period from 1975 to 2022 to identify references describing health systems development in the MENA region. The review process also included grey literature, official reports, policy and planning documents, and online resources by international organizations such as the World Bank and WHO. The literature review was conducted using keywords related to the topic including health reform, health system, development, improvement, performance, health financing, health outcomes, Middle East and North Africa region, MENA, MENA countries, and by applying different combinations of these keywords. At the outset of the review process, all relevant titles, abstracts and resources were identified, then they were filtered to exclude irrelevant references. During the next review cycle, the identified references were examined to select the references that would suit inclusion and detailed examination. The references were included in the review if they were describing the MENA healthcare systems with a focus on health system reforms and challenges facing these systems. All included references were in English language. The retrieved references were reviewed to extract, analyze, organize and report the findings.

## Overview on health systems organization in the MENA region

The health systems in most MENA countries were organized to provide primary health care services (4, 21). More recently, there has been a general trend toward curative care with large investments in acute hospital care (3–5). This has been driven by the health care and demographic changes that were accompanied by the growing burden of chronic diseases (4).

The state has been mainly responsible for the provision of health services in most MENA countries, *via* processes characterized by centralized financing, delivery and regulatory infrastructure (4). However, increasing attempts have been made by many governments in the region to separate these functions to help increase the efficiency and effectiveness of the health sector (4). These governments have also been shifting the delivery of health service to independent management systems for the operation of the primary and acute health care facilities (4). There has been an ongoing growth of national health insurance programs characterized by tiered coverage levels and different resources in several countries in the MENA region (3–5, 16, 22). This has been accompanied by the growth of the private medical sector to fill gaps in health delivery coverage, with subsequent concerns about efficiency, equity and quality assurance because of lack of proper control of this sector by some governments (3, 4, 23). This sector is currently playing a key role in the health care delivery in some MENA countries, specifically the GCC countries (4, 16).

In low-income countries of the region, two-tiered health systems that provide health services by both government and private sectors have been developed and implemented (4). However, the quality of the provided health services has often been suboptimal due to poorly trained staff, lack of availability of medications and medical supplies and inadequate coverage of the population needs in the rural and remote areas (4). This has reflected on an increase in the OOP payments to access private health services, an action that can be impoverishing for the poor (4).

The governments of middle-income MENA countries have been adopting health reforms targeting the organizational and financing aspects of health systems such as the implementation of social health insurance systems (3, 4, 22). This has resulted in an increased demands for different providers, including the voluntary and private (for-profit and non-profit) sectors to overcome gaps in population and service coverage, resulting in fragmented health care delivery and financing systems (4).

The populations of the upper-income countries in the MENA region have been provided comprehensive health coverage, either free of charge or at highly subsidized rates as a result of significant oil revenues (4). However, the provided health services in these countries could benefit further from improved efficiency and quality reforms (4, 13). The GCC governments had to implement cost-containment measures and as a result, have been looking for new financing strategies, such as introducing national health insurance schemes (4, 16). Moreover, most of the GCC countries have developed visionary policies. For example, Qatar had a National Vision 2020; similarly Oman prepared Vision 2020 followed by Vision 2040; the Saudi Arabia, Kuwait, the United Arab Emirates and Bahrain each prepared a Vision 2030 for sustainable development (24). These policies call for diversify of the economy, industrialization, and an emphasis on the role of the private sector in the delivery of healthcare services and the investment in workforce capacity building (24).

## Health patterns, disease burden and population dynamics in MENA region

Available evidence indicates significant improvements in key health indicators including a rise in life expectancy, decline in infant and maternal mortality, and expansion in health coverage, albeit to variable degrees in all the countries of the MENA region (3, 4, 11, 14). Table 1 summarizes selected health indicators for countries in the MENA region (25).

**Table 1:** Selected health indicators for some countries in the Middle East and North Africa region^a^.

	**Life expectancy at birth (years) (2019)**	**Adult mortality rate^b^ (both sexes) (2016)**	**Infant mortality rate^c^ (both sexes) (2020)**	**Hospital beds (per 10,000 population) (2017)**	**Medical doctors** **(per 10,000 population)**	**Nursing and midwifery personnel** **(per 10,000 population)**
Algeria	77.13	95.03	19.46 (18.47–20.52)	19 (2015)	17.19 (2018)	15.48 (2018)
Bahrain	75.81	56.81	5.78 (4.34–7.73)	17.40	9.26 (2015)	24.94 (2015)
Djibouti	65.81	244.50	47.18 (28.17–76.07)	14.00	2.24 (2014)	7.29 (2014)
Egypt, Arab Republic	71.82	164.60	16.65 (11.31–24.23)	14.30	7.46 (2019)	19.26 (2018)
Iran, Islamic Republic	77.35	80.13	11.14 (6.53–18.92)	15.60	15.84 (2018)	20.77 (2018)
Iraq	72.42	173.50	21.32 (16.4–27.48)	13.20	9.66 (2020)	23.87 (2020)
Jordan	77.87	110.50	12.92 (9.35–17.66)	14.70	26.61 (2019)	33.47 (2019)
Kuwait	80.97	79.22	7.58 (7.04–8.16)	20.40	23.42 (2020)	46.83 (2020)
Lebanon	76.44	95.62	5.97 (2.77–12.04)	27.30	22.07 (2019)	16.74 (2018)
Libya	75.78	150.30	9.53 (5.58–16.16)	32.00	20.91 (2017)	65.31 (2017)
Morocco	72.99	69.06	16.02 (11.36–21.85)	10.00	7.31 (2017)	13.89 (2017)
Oman	73.90	96.25	9.45 (8.38–10.68)	14.70	17.74 (2020)	39.38 (2020)
Qatar	77.17	61.76	4.93 (4.42–5.5)	12.50	24.85 (2018)	71.97 (2018)
Saudi Arabia	74.31	89.13	5.99 (4.61–7.94)	22.40	27.38 (2020)	58.17 (2019)
Sudan	69.15	223.90	39.92 (29.96–53.04)	7.40	2.62 (2017)	11.46 (2018)
Syrian Arab Republic	72.67	301.10	18.45 (9.58–24.45)	14.00	12.87 (2016)	15.41 (2016)
Tunisia	77.04	91.00	14.29 (12.64–16.09)	21.80	13.03 (2017)	25.14 (2017)
United Arab Emirates	76.08	73.95	5.62 (4.95–6.4)	13.80	26.01 (2019)	57.46 (2019)
Yemen, Republic	66.63	221.30	45.71 (23.97–81.09)	7.10	5.25 (2014)	7.85 (2018)

a Most recent data from the World Health Organization Global Health Observatory Indicators Index Data. Source of data: (25).

b Adult mortality rate is defined as the probability of dying between 15 and 60 years per 1,000 population.

c Infant mortality rate between birth and 11 months per 1,000 live births.

There has been an epidemiological transition that is manifested as a decline of the high burden of communicable diseases and an increase in the burden of injuries and non-communicable diseases (NCDs), but at a varying pace and timing among the different courtiers in the region (3, 20). Cardiovascular diseases, cancer and diabetes account for almost one third of the overall disease burden in the MENA region (26). The prevalence of risk factors for NCDs (obesity, raised blood glucose, hypertension and smoking) is far higher than the worldwide averages (11). The rising rate of smoking and tobacco product use is among the significant public health concerns in the region (4, 11). There has been an accelerated increase in obesity rates and tobacco smoking especially among women and adolescents which have been contributing to an accelerated rise in the prevalence NCDs (13). The main cause of mortality in the MENA region has been heart diseases which are anticipated to contribute to about 77% of total deaths by 2030 (13).

Some of the MENA countries such as the low-income countries and rural areas in middle-income countries (e.g., Egypt and Morocco) are challenged by dual burdens of disease characterized by increasing rates of NCDs accompanied by decreasing, but prevalent communicable diseases (4). An earlier study reported that NCDs and injuries are the underlying cause for more than 75% of the disability-adjusted life years in most lower middle-income countries except in Sudan and Yemen (11). In these two countries, NCDs account for less than 50% of the disease burden while infectious and parasitic diseases comprise a significant share (11).

On the other hand, middle- and upper-income countries in MENA are mainly impacted by the burden of NCDs while having largely eradicated communicable diseases (4). Cardiovascular disorders, diabetes mellitus, behavioral and mental disorders and malignant neoplasms account for more than 60% of the NCDs disease burden in most of the countries in the region (11). The rapid rise in NCDs is attributed to the rapid urbanization and changes in diet and lifestyle of the population of these countries (4).

The MENA region is also experiencing a high prevalence of stunting as a result of undernutrition, especially in low-income countries and some territories of middle-to high-income countries (4). There is also the prevalence of iron-deficiency anemia and other micronutrient deficiencies (4). Another major challenge is the increasing prevalence of overweight and obesity as a result of over-nutrition and sedentary lifestyle and their links to NCDs, especially in high-income countries in the region (4, 11, 13).

Another major cause of premature mortality in the MENA region is the increasing numbers of road traffic injuries which show no signs of reduction (4). This has mainly resulted from increased rates of urbanization and traffic volume in the presence of inadequate road infrastructure and safety measures (13). This has been estimated to account for the third top cause of mortality in the region and is expected to increase between 30 and 40 % by 2030 if no measures are implemented to change the existing policies (13).

Similar to other countries in the world, the MENA region has been facing emerging diseases such as HIV/AIDS, Middle East Respiratory Syndrome (MERS-CoV) and the Coronavirus Disease of 2019 (COVID-19) pandemic (4, 27, 28). The overall prevalence of HIV/AIDS is still low and limited to high-risk groups (4). However, it has been estimated that the MENA region has the second top growth rate of HIV infection in the world (4). This necessitates timely and effective preventive actions to be undertaken to combat the spread of the disease and limit its social and economic consequences (4).

The MENA region has been facing rapid expansion in the population (4). It had a 3.7-fold increase in population from 1950 to 2000, which was the highest population growth rate in the world (4). At present, it is estimated that MENA has the second top annual population growth globally (at 2% equivalent to about seven million additional individuals per year) (4). If this rate continues, the population is expected to double during the first half of the twenty first century (1, 4). The MENA countries vary widely with respect to their population size, with the highest population in Egypt (11). It is estimated that all MENA countries (except Lebanon) will witness a significant increase in the population during the next decades (1). The highest increase in the total population in absolute terms is expected to be seen in Egypt, followed by Iraq and Sudan (1). The population of some countries such as Iraq, Sudan and the State of Palestine are expected to double in size in the 30 years between 2015 and 2050 (1). In contrast, the population of Lebanon will decline by 480,000 by 2030 (1). Urbanization will continue to grow in many MENA courtiers such as Kuwait, Qatar, Jordan, Oman, Lebanon, Bahrain, and the United Arab Emirates (1).

The fertility rate in the region has been decreasing for years due to multiple factors such as delayed marriages and the use of contraception (1). It is estimated that the total fertility rates in half of the MENA countries will be at or less than the level of replacement by 2030 (1). However, a history of high fertility rate has yielded to an increasing number of women of reproductive age (1). This will lead to a larger number of births that is estimated to be 500 million births during the first half of the twenty first century (1). The MENA countries stand at various stages of the demographic transition, varying from countries in the early transition stage, characterized by both high birth and mortality rates, to countries that achieved the transition with both low birth and mortality rates (4).

The number of children and youth (0–24 years) is expected to decrease in all MENA countries (except in Iraq and Sudan) in 2050 (1). The number of youth will be increasing in 12 MENA countries, but will contribute to a declining proportion of the overall population due to decreasing fertility (1). As mortality and fertility rates decrease in most of these countries, the population's age structure changes (1). The MENA region is undergoing a demographic dividend which happens because of the demographic transitions of the population of its countries, albeit at different rates (1). The United Nations Children's Fund (UNICEF) has classified MENA countries into three categories in terms of demographic phase (1). Pre-dividend countries are those with an increasing proportion of working age population between 2015 and 2030, due to ongoing rapid increase in population growth leading to a high child dependency ratio (1). These include four countries, the State of Palestine, Iraq, Yemen and Sudan (1). The early-dividend countries which are experiencing a relative rise in the working age population and are in their way toward decreased fertility; thus witnessing lower child dependency ratios and a higher share of working age population (1). This applies to ten MENA countries and includes Egypt, Libya, Algeria, Iran, Jordan, Bahrain, Saudi Arabia, Oman, Syria and Djibouti (1). Late-dividend countries are those experiencing a decreasing proportion of working age population between 2015 and 2030 due to low fertility rates, but raising elderly-dependency ratio and include six countries: Kuwait, Qatar, Tunisia, Morocco, Lebanon and the United Arab Emirates (1).

In the MENA region, LEB has increased dramatically since the 1950s and is expected to witness further increase during the coming decades, but at a slower rate and varying degrees among the different countries (1, 11). In countries like Lebanon, Morocco, Algeria, Iran, Tunisia, Oman, Qatar, Bahrain and the United Arab Emirates, the life expectancy is expected to increase to more than 80 years by 2050 (1). On the other hand, Djibouti, Yemen and Sudan are expected to have the lowest projected LEB which will be ~70 years in 2050 (1).

An important contributor to the population dynamics in the MENA region is the migration to, from, and within the region (4). The MENA countries have been affected by migration and forced displacement as a result of conflict and war, as well as for employment and climate-related migration (1). The World Bank classified eight MENA countries as being conflict-affected or fragile contexts in 2018 (1). The MENA region also hosts the largest refugee population in the world (4). In 2010-2011, several MENA countries have witnessed a wave of mass uprisings over poverty, unemployment, lack of justice and political repression, the so called the Arab Spring (3). The main driver for the mass protests was a call for social justice, wellbeing and dignified life for all constituents (16). As a result, several MENA countries including Libya, Syria, Iraq and Yemen have been suffering from continued violent conflicts (3). This has created regional refugee crises and sustained impacts and pressures on the health systems of the countries that have been hosting the refugees such as Lebanon and Jordan (1, 3, 29). These include limited access to health services for the refugees who have chronic NCDs, rise in conflict-related injuries, destruction of health infrastructure, migration of most health workforce and outbreaks of infectious diseases (3). Lebanon, Gaza and the West Bank witness political instability and Iran is still experiencing world-wide banding measures (3). More stable countries such as Algeria, Morocco, Tunisia and Jordan have been also undergoing significant reforms (3). On the other hand, the oil producing GCC countries have been challenged by the significant influence of the economic migrants on their population size and structure (1, 4). These countries have been hosting millions of expatriates, constituting between 60 and 90% of their workforce since the exploration of oil in the 1970s (4). This has added responsibilities on the GCC countries to provide healthcare services to meet the health needs of the workers and their families (4).

## Challenges facing health systems in the MENA region

According to the WHO, MENA countries will have to address several weak aspects of their health systems including weak policy analysis, formulation, coordination and regulation, limited cooperation among sectors, poor community participation in planning and provision, and inadequate health information systems, human resource policies and management of health services at all levels (4). Poor planning has resulted in inefficient and misuse of resources and consequently led to the growth of unregulated markets, decreased productivity and increased the pressure on limited financial and human resources (5).

In the MENA region, health care planners and providers have been facing major challenges (5). These include increasing pressure on health services as a result of growing demands due to demographic changes, technological advances and rising public expectations, augmented by economic sanctions in some countries (5). As stated earlier, the MENA healthcare systems have also been challenged by the growing burden of NCDs and a combined burden of malnutrition that involves both undernutrition and obesity (16). The increasing NCDs along with their social, economic and political implications have been among the significant challenges that have been hindering countries' effort to achieve UHC (30).

The MENA countries face challenges related to the financing needed for the provision of pharmaceuticals and medical equipment (4). Lack of properly functioning pharmaceutical regulations, inappropriate medication prescribing and self-medication practices contribute to a high percentage of THE on pharmaceuticals (4). These countries largely depend on passive purchasing of health products instead of adopting active purchasing provider payment mechanisms which can enhance quality, productivity and equity (3). Expenditure on medical equipment and technology is also inefficient and excessive in view of the predominance of the curative care approach in the health systems in the MENA region (4). Many MENA countries adopt curative approaches, especially for NCDs management, rather than adopting preventive approaches, which leads to increased health care costs (3).

Another challenge is related to the legacy of health services reforms in the MENA countries which have been mainly financially driven instead of being need-driven (5). This has resulted in accelerated and disorganized expansion of medical services provided by the private sector, with a main emphasis on curative care than on preventive medicine or the wider public health agenda leading to further pressure on the limited available resources (5). Additionally, the regulation, monitoring and control of the private sector is uneven and limited in several MENA countries (3). However, some countries such as Egypt, Jordan and Tunisia have public or independent accreditation bodies for the public and private health facilities (3).

Among the prominent challenges facing the MENA region is the inequalities in health status which is characterized by wide variations in population health status among the MENA countries, and sometimes within countries due to unequal distribution of resources, variations in the population socioeconomic levels and inappropriate planning to target the most deprived groups of the population (5). Health infrastructure and services are mainly localized in urban areas in most of the MENA countries, reflecting the general distribution of the population (3). There is unequal access to care, a wide variation in access to health services across and within countries with large uncovered population in several countries and territories in the MENA region (3, 16). There have also been issues related to procurement, importation and provision of critical medical technologies, products, vaccines and other technologies in many countries in the MENA region due to the geographic adversity within some of these countries and existence of rural population living across an enormous geographic territories (4).

Many of these countries also face challenges in training and recruiting healthcare workers, especially in conflict settings and rural areas (3). In addition, variable and inconsistent quality and long waiting times with high absence rates among healthcare workers have been among the issues that face some healthcare systems in the region (16). Other challenges include the wide variation in the clinical standards and shortage of valid and reliable data on health systems performance (5). The MENA region generally lacks available, timely and reliable data to support conducting adequate health reforms (3, 4). Among the leadership challenges facing the MENA health systems include poor development of leadership skills, limited support beyond the top-most level of leadership, lack of comprehensive information for management and existence of competing interests posed by various stakeholders in these system (4).

These challenges have been coupled with the ongoing conflicts in several countries of the MENA region which have led to political instability, declined economic productivity and large decrease in governmental incomes (16). Providing adequate funding for health services for refugees is another main concern in the countries that host large refugees population (3).

## Health workforce

Healthcare professionals represent the core of the health system (10). Poorly qualified health workforce is a commonly recognized problem (5). The main challenges facing MENA countries in terms of health workforce include the recruitment and retention of the properly trained workforce, professional licensing and continuous education and recertification for all health professionals, and the need to provide strong organizational culture with clear standards and policies to support a diverse health workforce needs (4).

The health systems in the MENA region are impacted by the universal challenges of preparing, maintaining and retaining health professionals along with a considerable variation in the human resource situation among and within countries of the region (4). A study in 2017 has found that the mean physician density in the MENA region (20 physicians per 10,000 population) is superior to the worldwide physician density of 13.9 (11). The region has also been found to have higher nursing and midwifery staff density (36.8 vs. 28.6 per 10,000 population) compared to the global averages (11). However, physicians and nursing density in many MENA countries was found to be lower compared to the averages of countries with comparable income and a decline in physician densities was detected in several countries in the region (11). The majority of the health staff practice in urban areas while rural areas often lack adequate number of well-equipped health personnel (4). In addition, there is a shortage of female health workers in MENA countries, which can be a main access issue as female staff are needed to attend to female patients due to cultural beliefs in these countries (4). This could reflect lack of adequate policy and regulations to support development and adequate distribution of healthcare professionals in MENA region (11).

The health workforce of the MENA region vary widely (4). This is evident in the disparities in the number of physicians per 1000 population among MENA countries (Table 1) (4, 25). Low-income countries in MENA experience a lack of employment, deployment and retention strategies for health workforce, migration of competent health professionals (brain drain), and shortage of reliable information on human resources for health which hinders proper decision-making and policy formulation (2). The GCC countries, which rely heavily on expatriate medical staff have been focusing attention on motivating their citizens to receive education and training to serve in the healthcare sectors, while launching educational frameworks to guarantee the development of their professional skills (31).

## Financing of health systems in the MENA region

Health financing encompasses all the resources that can be collected and utilized to improve the population health status and to protect the individuals within this population from risk factors that may harm their mental, physical and social development (3, 17). Financing describes the methods by which money is raised, risk pooled and billed to support changing the operation of a health sector (16, 17). Financial protection, which is an important health system outcome can be assessed by examining OOP spending on health services (15, 17). This in turn can be evaluated using different ways ranging from estimating the number of individuals that either fall into poverty or decline in the level of poverty because of OOP spending on healthcare to a simpler measure of the OOP spending on healthcare as a percentage out of total health spending (15). High OOP would indicate an increased financial risk on the population more than low OOP (15).

In general, countries of the MENA region have some of the lowest percentages of public expenditure on health when compared to other countries in the world, which is reflected as high levels of OOP expenses (16). The public spending in the MENA region has been low, representing about 3.2% of GDP in 2014 (16). The general government health expenditure (GGHE) as a percentage of general government expenditure (GGE), an indicator which reflects the priority of the government to spend on health was reported to be 7.0–8.0% on average in the period between 2000 and 2014 (11). This has been further diminished because of the political instability and fluctuation in the price of oil and gas in some of the region's countries (16). This has obliged many individuals living in these countries to either miss receiving adequate care or face hardship due to health expenses (16). Uncovered household expenditure on health care is still high in many countries in the region resulting in high levels of OOP household spending (3).

MENA countries, at the all income levels, have been spending a comparatively less percentage of their income on health represented as a percentage of GDP (13). There is also a wide variation of health care expenditure as percentage of GDP within the countries in the region (12). Table 2 shows some key health expenditure statistics for countries in the MENA region (25, 32). Along with that, public spending on healthcare as a percentage of total government expenditure has been lower as compared to other countries in the world, while private OOP spending being high (2, 11, 13). It has been shown that MENA countries spend less on health care when compared to high income countries (12). This could mean that the governments of the region have accomplished costs-containment and perhaps an efficient delivery of health services (13). However, there is an accumulative evidence suggesting quality issues and inefficiencies in many healthcare systems in the region (13). Examples include countries such as Egypt, Morocco, Tunisia and Yemen which reported considerable rise in OOP spending as a percentage of total health spending (13). The high income GCC countries have been able to extend coverage to their residents in view of their considerable fiscal space (13). However, questions have been raised around the quality of care and efficiency of utilizing the available resources in these countries (13).

**Table 2:** Key health expenditure statistics for some countries in the Middle East and North Africa region^a^.

	**GDP US$ per capita** **(2019)**	**Current health expenditure (CHE) per capita^b^ in US$** **(2019)**	**Current health expenditure (CHE) as % of gross domestic product^c^ (GDP) (%)** **(2019)**	**Government health spending as % of current health expenditure^d^ (CHE) (%)** **(2019)**	**Out-of-pocket expenditure as % of current health expenditure^e^ (CHE) (%)** **(2019)**	**Priority to health (GGHE-D%GGE)^f^** **(2019)**
Algeria	3,976	248.20	6.24	65.00	33.44	10.73
Bahrain	23,443	940.40	4.01	59.20	29.73	7.23
Djibouti	3,437	61.81	1.80	53.70	24.15	4.28
Egypt, Arab Republic	3,161	149.80	4.74	27.80	62.75	4.66
Iran, Islamic Republic	7,010	470.40	6.71	49.50	39.49	21.40
Iraq	5,568	253.30	4.48	49.40	50.10	5.99
Jordan	4,405	334.00	7.58	51.20	30.29	12.80
Kuwait	31,999	1759.00	5.50	87.00	11.79	8.93
Lebanon	7,668	663.10	8.65	49.00	33.54	13.43
Libya	9,337 (2006)	309.90 (2011)	6.05 (2011)	65.40 (2006)	36.67 (2011)	6.42 (2011)
Morocco	3,282	174.20	5.31	39.90	46.81	7.12
Oman	15,343	624.70	4.07	86.40	6.56	7.98
Qatar	62,088	1807.00	2.91	72.80	12.33	6.50
Saudi Arabia	23,140	1316.00	5.69	69.20	16.50	11.05
Sudan	1,026	46.93	4.57	22.70	67.38	5.56
Syrian Arab Republic	1,958 (2012)	69.83 (2012)	3.57 (2012)	45.30 (2012)	53.69 (2012)	4.47 (2012)
Tunisia	3,349	233.10	6.96	57.10	37.94	12.58
United Arab Emirates	43,103	1843.00	4.28	52.30	12.51	7.40
Yemen, Republic	1,446 (2012)	73.18 (2015)	4.25 (2015)	23.90 (2012)	80.96 (2015)	2.23 (2015)

a Most recent data from the World Health Organization Global Health Observatory Indicators Index Data and the Global Health Expenditure Database. The data is for the year 2019 unless indicated otherwise next to the value. Source of data: (25, 32).

b Per capita current expenditures on health expressed in respective currency—US dollar.

c The level of current health expenditure expressed as a percentage of GDP.

d The share of governmental health spending of total current health expenditures.

e The share of out-of-pocket payments of total current health expenditures.

f Domestic general government health expenditure (GGHE-D) as percentage of general government expenditure (GGE) (%): is the share of general government expenditures funding current health expenditures.

Overall, there has been a trend toward increased health expenditure in all non-fragile MENA countries in per capita terms and as a percentage of public spending or as share of the income of a country (3). Asbu et al. (11) reported that per capita total expenditure on health has increased, albeit with a wide difference among the various income groupings in the MENA region. They indicated that total expenditure on health as a share of GDP has slightly increased over the years but the health system seems to constitute a small share of the economies of most countries in the MENA region (11). Their analysis of health systems financing in the MENA region revealed that the total health expenditure (THE) per capita in most of the MENA countries was lower than the averages for countries under comparable income category in the world (11). Additionally, the increase rate of THE per capita has not been correlating with the growth rate of GDP per capita (11). With the exception of the high-income countries in the group, OOP spending in MENA countries exceeds the limit for catastrophic spending indicating that households are at an increased risk of suffering poverty as a consequence of spending on healthcare (11). Their results showed that most MENA countries depend on OOP spending which ranged from as low as 6% in Oman to 76% in Yemen and Sudan in 2014 (11). The GCC countries were found to have lower OOP spending on health services than other MENA countries (11).

Overall, there has been a tendency toward spending a greater share of the GDP on health in the higher-income MENA countries (3). In a study assessing the impact of healthcare expenditures on healthcare outcomes in the MENA region between 1995 to 2015, Balkhi et al. (12) found that spending on healthcare has been rising for many countries in the region, with higher spending per capita on health in Kuwait and the United Arab Emirates than any other countries in the region. Their findings showed a correlation between health expenditures and health outcomes across different countries in the region, yet some countries were found to have shorter life expectancy despite spending more on healthcare (12). Accordingly, they recommended for effective and efficient use of healthcare resources as a potential approach to improve health outcomes in any country (12). Other studies have shown the positive impacts of increasing health spending in MENA countries on the health outcomes of the population including improving LEB and reducing mortality rate (12, 33).

There have been wide variations in the access to health services and the coverage of health care for patients with NCDs across and within MNEA countries, with some countries providing public insurance schemes (3). However, patients who lack such coverage have limited financing solutions aside from OOP expenses (3). Elgazzar et al. (22) examined the scope of OOP expenditures and their impacts on policy reforms and living standards in six MENA countries in 2010. Their analysis showed that OOP spending was 49% on average in the MENA region, which represented a relatively high percent of total national health care financing with households spending about 6% of their total household expenditure on health mainly on medications, physician visits and diagnostic services (22). They identified the persistence of catastrophic health spending in MENA region despite the availability of services and insurance schemes directly provided by the governments with an incidence that is relatively high in comparison with similar low- and middle-income countries (22). Over time, OOP expenditure on healthcare services can become catastrophic with significant implications on the living standards of people (22). The WHO defines financial catastrophe as the situation when “direct OOP payment exceeds 40% of household income net of subsistence needs” (34).

The private sector seems to have a limited role in providing and financing of health care in the MENA region with a limited private insurance market and most of the private spending originating from direct household OOP spending at the point of services (13). A limited number of health reform initiatives in the region have been focused on the expansion of contracting of private providers directly by the Ministries of Health or by social insurance funds (13). Examples include health reforms tackling this issue in Lebanon and the Palestinian Authority (13, 35).

Reforming health-financing systems is essential to achieving UHC, which has been endorsed by the WHO to help create or build health equity through financial protection (36). MENA governments have been actively working to extend financial protection and enhance access to health services by utilizing various risk-pooling mechanisms, including the development of private and social health insurance (4). Providing well-targeted social safety nets can help protect citizens against the depleting consequences of ill health (4). Indeed, most MENA countries are low- and middle-income countries with very limited fiscal space to allocate the financial resources to meet the expected increase in the demands for health care (13). Therefore, efforts have been made by some of these countries to expand the revenue base and improve the risk pooling to cover the additional resource requirements (13). For example, Egypt, Algeria, Morocco, Tunisia and Jordan have begun to adopt new health policies and strategies to address health financing in response to substantial health system challenges including large uncovered population and an inconsistent quality of healthcare services (3).

Among the options that have been considered and tested by some of the countries in the MENA region include: (1) introducing new, or expanding the existing, social health insurance schemes; (2) improving the regulation and organization of the private health insurance sector to help households to utilize their OOP spending on health for the purchase of a prepayment program; and (3) introducing taxes on certain services and goods that have direct impacts on public health to broaden the revenue base for health care (13). There has been a slow increase in prepayment schemes of health expenditure as health insurance and tax-based schemes in the region (3). In addition, there has been limited external support, such as receiving funds and assistance from international organizations and donors to some of these countries, mostly as budget support and specific funding for some projects (3). The health reforms initiatives in many countries in the MENA region can have an essential role in improving the health outcomes of the population of these countries while maintaining health-related costs (37).

## Examples on health systems reform in the MENA region

Several countries in the MENA region have been adopting and implementing health reforms targeting different aspects of health system performance. The examples presented in this section do not comprise an inclusive overview or evaluation of health system reforms across all MENA countries but provide examples of such reforms and some country experiences during the last years. The presented examples are outlined in light of the Flagship Framework that has been adopted to guide health system reform (17).

In 2017, a special issue of Health Systems and Reform Journal encompassed a set of articles on health system reforms in some MENA countries which were supported by the World Bank to address health system needs and to meet the demand of their population for better health care equity, access and delivery (16). In this issue, Wang and Yazbeck examined and performed benchmarking of the health systems in the MENA region in terms of health status and financial protection, two of the three designated performance goals of the Flagship Framework (15–17). Their findings revealed wide vibrations in these outcomes across MENA countries even though these countries share considerable linguistic and cultural similarities (15). This article reported evidence supporting the varied health outcomes among these countries (15). They compared LEB between MENA countries and used it as a measure of health status (15). Their findings showed that there seem to be no strong relationship between Per capita national income and LEB in these countries (15). They found that Morocco, Lebanon, Algeria and Tunisia performed best on LEB after controlling for per capita national income and educational stock (15). They also found that most GCC countries, especially Qatar, Kuwait, the United Arab Emirates and Saudi Arabia were underperforming on LEB relative to their level of development and were also underspending on health (15).

They classified the countries into high and low performing health systems (15). With regards to health status, this article identified three categories of countries with certain trends in their levels of national income and educational stock, both of which are highly related to health outcomes (15). The three categories are: (1) countries with under-expenditure on health, sometimes significantly, along with underperformance in life expectancy; (2) countries with slightly better than anticipated health expenditure but significantly higher than anticipated life expectancy; and (3) countries with marginally lower health expenditure and slightly higher than anticipated health status (15). They identified Iran, Egypt and Bahrain as countries that could achieve good health outcomes at a reasonably low spending, indicating that their health systems were efficient in utilizing the available resources (15). Their results showed that Morocco, which was the best performer on LEB exhibited the top percentage of OOP among the MENA countries (15). On the other hand, the GCC countries had the lowest percentage of OOP spending among all MENA countries but they did not perform well on the LEB compared to national income and educational stock (15). They concluded that within the MENA region, those countries with less performance in terms of financial protection (e.g., Egypt and Morocco) achieved better performance in terms of health status outcomes (15). Few countries such as Lebanon and Bahrain could achieve good health outcomes comparative to income and education with relatively good performance in providing financial protection (15).

In another article, Pande, El Shalakani and Hamed assessed the situation of the health system in Egypt and proposed a novel diagnostic tool to assess improvement toward achieving social justice in health care using the three performance goals defined by the Flagship Framework (7, 16, 17). They performed a thorough analysis of primary and secondary quantitative and qualitative data sources and identified six disadvantaged groupings in Egypt (7). These included households in the lowest wealth quintile, population resident in certain geographic areas, population with low parental education, workers in the informal sector who lack coverage by insurance schemes, women and disabled people (7). Then, they analyzed the status of these groups in terms of the three performance goals of a health system which include improving health outcomes, public satisfaction and financial risk protection (7). Their findings indicated that there were 11 challenges to the achievement of social justice in health care in Egypt (7). These included high OOP spending, poor quality of care in certain sub populations, rising burden of NCDs, and poor maternal and child health indicators (7). Accordingly, they suggested a number of short- and medium-term recommendations to address these challenges and advance social justice in health care in Egypt (7). These included recommendations to improve the health of disadvantaged groups such as introducing a family health services model of primary care, recommendations to enhance financial protection for disadvantaged groups such as the separation of providers from payers and recommendations to enhance the quality of health care such as introducing opportunities for the participation of citizens in service delivery (7).

Al-Mazrou et al. (23) described how the private health insurance for expatriates developed following a labor law was passed and how this influenced the financing, organization and delivery of health services in Saudi Arabia. This reform was directed toward the financing and payment knobs of the Flagship Framework (16, 17). Similar to other GCC countries, Saudi Arabia has been changing the financing and delivery of health care partly due to the high proportion of expatriate workers in the country (23). A labor law was implemented in 1999 which obliged the private employers to guarantee that expatriate workers have health care coverage by private health insurance instead of depending on the public health system (23). This labor law resulted in an expansion in the insured population with a steady progress in private health insurance market which became the primary funder for the private health sector (23). This resulted in a significant growth in the private hospital sector with an increase in the available private providers and private hospitals in urban areas (23). It also changed the payment methods of hospitals and other facilities (23). This experience demonstrates how the health systems can be influenced by policy reforms (23).

In Lebanon, the main provider of hospital care and contractor to the Ministry of Public Health for providing curative care is the private sector (35). Khalife et al. (35) described the experience of Lebanon in contracting reform and the development of a provider payment from the public sector to the private hospital sector and how this approach produced positive outcomes for the health system. This reform experience targeted the payment knob of the Flagship Framework (16, 17). A mixed-model hospital contracting model was implemented in 2014 (35). The reform program involved adopting several developmental strategies including the use of an automated billing system and a utilization review, and development of standardized criteria for admission and a hospital case mix index that accounts for the complexity of cases (35). This experience fits well with a regional strategy that is focused on developing a stronger partnership between public and private sectors and the development of the private sector to provide hospital care (35). The authors outlined important lessons from their experience about methods for provider payment, and how to link payments to different quality measures including accreditation, data management and effective use of information technology (IT), community engagement and the nature of public–private collaboration (35).

In another article, Alaref et al. (38) assessed the consequences of an organizational reform on the quality of care in Palestine. They examined the possible implications of prohibiting dual practice (defined as the practice of a health professional concurrently in both the public and private health sectors) on the access to, and quality of health services (38). This represented a case of reform that adjusted the organization knob of the Flagship Framework by focusing on policies to address dual practice among physicians (16, 17, 38). Based on their analysis, they outlined that a complete ban on dual practice would lead to several adverse consequences including the risk of losing the rare specialties from the public sector (38). They indicated that such policy would not decrease the fiscal burden on patients nor, does it improve their access to quality health services in the public sector in Palestine (38). They proposed a set of recommendations to tackle this issue including the need to conduct several studies, monitoring policy implications and provide incentives for health workers (38).

Le Pape et al. (39) reported the experience of the health sector in Morocco in adopting and implementing information and communications technology to enhance the productivity and effectiveness of the sector. This is another example on a reform focusing on the organization knop of the Flagship Framework which examined the role of IT on the organization and function of the health sector (16, 17, 39). This organizational reform experience involved the establishment of a national health management information system in Morocco and showed how an effective information system can facilitate decision making at all levels of the health system (39). The authors recommended engaging all stakeholders from inception to implementation, promoting local ownership of the new system, implementing personnel rotation policies and supporting capacity building and staff upskilling efforts (39).

Several other reports on different experiences of health reforms have been emerging from the MENA region. An important direction in terms of health reforms in the region includes reforming or establishing health insurance systems to generate money for the health sector and to pool risk across the population (16). Examples of countries that have been adopting such strategies are the GCC countries (16). The government in Morocco has also undertaken a health finance reform supported by the World Bank and the European Union (5). The goals of this project were to improve equity in the health system and ensure sustainable and stable financing (5). This project required every individual who has an income to subscribe to a health insurance scheme (contributive system), while subsidizing those who are financially weak by creating a medical assistance mechanism (5). This would allow greater collective financing of health services and a reduction in the OOP payments by households, which represent a major cause for inequity (5). Similarly, in Tunisia, the government attempted to apply an insurance system that would cover the provision of health services by the private sector (5). These efforts can be viewed as health reforms targeting the financing and payment knobs of the Flagship Framework (16, 17).

Alhuwail (40) documented positive improvements in the compliance of the secondary care public hospitals in Kuwait with the information management accreditation standards. These included improvements in data privacy and security, transfer of information, data aggregation to support patient care, analytics for decision-making, information exchange, staff access to internet and identification of quality performance indicators (40).

In Saudi Arabia, the healthcare system has been undergoing several developments driven by the Saudi Vision 2030 (21). A comprehensive framework for addressing public health challenges through adopting the concept of primary health care services has been developed and implemented in the country (5). This initiative targeted the organization knob in the Flagship Framework (16, 17). Several studies have reported the public healthcare system and primary health care reforms in Saudi Arabia and provided a review of the reform process, challenges facing the development process and recommendations to overcome these challenges (9, 21). There has also been an increasing trend toward privatization of the health care sector (41). Privatization of health care involves increasing engagement of non-governmental actors in providing healthcare services *via* management and finance services (42). In 1999, the Saudi government implemented a reform in its health policy and adopted a privatization strategy of health services to improve these services and to keep pace with the advancement of medicine, leading the private sector to flourish in the country (41). This strategy can be considered as a reform affecting the financing and organization knobs of the Flagship Framework (16, 17).

As an example of a health reform targeting behavior of patients, the GCC countries have been implementing a comprehensive plan against tobacco (5). This plan included the following initiatives: raising taxes on tobacco and its derivatives, imposing low levels of tar and nicotine in cigarettes, banning shisha in some of the Emirates, implementing a program called “Quit and Win” that provides incentives to encourage smokers to quit smoking, organizing free clinics to help smokers quit, organizing a national control and prevention week and anti-smoking campaign in schools, and conducting research to estimate the prevalence rates of smoking in schools (5). This can be considered as a reform targeting the behavior Knob of the Flagship Framework (16, 17).

Braithwaite et al. provided an overview of case-study accomplishments related to health system reforms from different countries in their textbook “Health Systems Improvement Across the Globe: Success Stories from 60 Countries” and in a subsequent article in which they commented on these success stories (18, 19). Their textbook included several examples of health reform experiences, case studies and health systems development efforts that were accomplished in some MENA countries including Oman and the United Arab Emirates (health reforms involving improved data and IT infrastructure such as the implementation of electronic health records), Jordan (a reform involving enhanced accreditation and regulatory standards) and Qatar (developing “Qatar Early Warning System” for deteriorating patients) (18, 19). Other examples included the experience of the GCC countries in the procurement of pharmaceuticals and medical supplies from other GCC countries, Iran (the comprehensive reforms *via* the health transformation plan), Yemen (an initiative directed to improving basic health services), and Lebanon (social innovation and blood donations initiatives) (18, 19).

In a subsequent textbook entitled: “Healthcare Systems: Future Predictions for Global Care”, the authors presented the experiences of 152 countries in health system improvement which were then summarized in an article (10, 43). Further examples of health system reforms from the MENA countries were presented in these publications (10, 43). These included: the experience from Iran in hospital accreditation, a human development initiative involving developing national human resources for health strategy in Jordan, and development of m-Health for healthcare delivery reform in Lebanon (10, 43). Other examples included a reform involving patients empowerment in Oman, a reform influencing hospital palliative care in Qatar, development of a national medical record in the United Arab Emirates, and the experience of Yemen in integrating public health and primary care services (10, 43).

## External support for health systems reform in the MENA region

The World Bank has been providing support to several governments in the MENA region to establish fair and sustainable health systems that can be transparent and accountable to the population of these countries (2). This has been achieved through providing expert advice and by financially supporting policies, reforms, processes, mechanisms, and tools that support transparency and quality of health care (2). It has also been supporting government initiatives aimed at enhancing the quality of healthcare services provided to the citizens, as actual purchasers at the point of service, or as tax or premium payers (2). The World Bank has been supporting initiatives to engage citizens and patients in deciding efficient ways for spending their money to provide more value and to become fully informed for active and purposeful engagement (2). It has also been providing countries financial and technical support in establishing health management information systems to help them improve accountability of payers (2). This can be mediated by decreasing costs through developing strategies aimed at promoting primary and preventive care, pharmaceutical reform, or addressing gaps in health care delivery (2). There has been increasing number of health system assessment and review reports describing the World Bank efforts in the support of health systems in different countries in the MENA region, especially middle- and low-income countries like Egypt, Morocco, Tunisia, Palestine and Yemen (44–48).

## Health accreditation in support of health systems reform in the MENA region

Health systems have been applying accreditation of healthcare facilities as a quality assurance method to ensure the delivery of healthcare services of adequate quality to patients (49, 50). Countries that seek to grant health accreditation have the goal of strengthening their health systems and improving patient safety either *via* national policies and standards, and/or accreditation (10).

Several countries of the MENA region have been implementing healthcare accreditation (51). For example, Lebanon developed its national healthcare accreditation program in 2002 as one of the earliest accreditation programs in the region (52). The Central Board for Accreditation of Healthcare Institutions in Saudi Arabia was founded in 2005 to create and evaluate the quality standards of all healthcare sectors, and the public and private hospitals in the country have additionally been seeking accreditation from international accreditation organizations (53, 54). There have also been efforts in obtaining international accreditation of healthcare organizations in Qatar (55, 56). Another example is Jordan, in which the Health Care Accreditation Council was founded in 2007 to assure the quality of care of healthcare organizations, and similar to Saudi Arabia health facilities have also been seeking accreditation from international accreditation bodies (57–59). In Kuwait, the Ministry of Health established a partnership with Accreditation Canada International in 2008 to develop a national health accreditation program for the development, implementation and evaluation of healthcare quality standards at all public healthcare care facilities (60).

## Health system responses to COVID-19 pandemic in the MENA region

The MENA region has been impacted by the COVID-19 pandemic in late 2019–early 2020. The social distancing measures such as workplace closures and lockdowns have reduced economic productivity and increased inequalities (28). This has mainly affected the most disadvantaged households who mainly work in informal jobs, lack health insurance, and are more susceptible to infectious diseases because of overcrowded living conditions (28). The risk of morbidity and mortality due to COVID-19 is also high in the MENA region due to the high burden of NCDs and a wide range of at-risk populations due to poverty, inequality and humanitarian crises (27).

The healthcare systems within the MENA region have been challenged by the COVID-19 pandemic which was a stress test for the resilience of these systems to meet the preexisting healthcare needs of the population (61). Before the pandemic, there have been underfunding for the public healthcare systems in MENA, particularly in countries with middle-income, even though the share of the public sector had grown as a portion of GDP (61). The pandemic revealed inadequacy of core elements of public health resilience which reflects the ability of healthcare systems to absorb the attacks of health emergencies, mainly the surveillance and absorptive capacity (61). Some countries such as the high-income GCC countries adapted and responded quickly to the pandemic and managed successful responses both on the policy and pandemic containment efforts (61). While many other countries failed to achieve such prompt responses or to make adequate emergency investments in their public healthcare systems (61). The COVID-19 will have a significant implication on the MENA health systems, both directly and indirectly by affecting care-seeking for other essential health services (27). This will augment the pre-existing limitations of the health systems such as the low and inequitable financing levels, fragmented and inflexible care delivery, limited and poorly distributed human and physical resources, and inadequate surveillance and health information systems (27).

It has been postulated that lack of data and data use may have contributed to the limited ability to provide a realistic assessment of the preparedness of the healthcare systems prior the pandemic in the MENA region (61). This has highlighted the value of effective use of data for public health policy making (61). The progress made in this aspect during the pandemic combined with a strong focus on building essential public health functions can be the basis for deeper health reforms and for constructing resilient healthcare systems in the region (61). Moreover, collaborative efforts are needed to support the necessary reforms to improve the level and distribution of health financing and to strengthen physical and human resources (27).

## Discussion

This review article provides an overview of the health systems organization, health patterns, disease burden and population dynamics in the MENA region. It also outlines the challenges that have been facing these systems and provides examples of health system reform experiences that were achieved in the countries of the region. Some of the reported reform experiences in these countries impacted the different elements of the Flagship Framework (16, 17). Some reform initiatives were directed to the fine-tuning of the control knobs of the framework including the financing (5, 23), organization (38, 39, 41), payment (23, 35), and behavior (5) knobs, or they evaluated the ultimate performance goals of the health systems (7, 15).

The challenges facing the MENA health systems are multifaceted at various levels and scope. These include health transition-related challenges such as the increasing pressure on health systems to keep pace with demographic and epidemiological transitions affecting the region including the rapidly growing population, rising prevalence of NCDs, increasing aging population and refugees' crises (4, 5). Other set of challenges are health-system related challenges which include challenges related to service delivery, financing, health workforce, medical technologies, information and leadership (3, 4). The governments of the MENA countries must engage all key stakeholders in the design, implementation and management of the health systems and in adopting adequate reforms to effectively and efficiently address these challenges (4).

In order to overcome the growing complex health and security challenges facing the MENA region, there must be a focus on developing effective health systems with effective health financing mechanisms to provide health services that are affordable, equitable and of adequate quality to all citizens, residents, refugees and displaced individuals (3). The aim of the financial policy reform in the MENA region must be directed toward increasing public revenues for the health sector to achieve UHC (11). To achieve this goal, governments must prioritize health on their agenda and allocate governmental budget to support healthcare services despite any financial constraints (62). In view of the growing population and high unemployment rate in many countries of the MENA region, government health budgets must be directed to supporting the needs of the most vulnerable populations to provide financial protection to these disadvantaged groups (4). To achieve reforms in healthcare financing systems, governments must adopt risk pooling mechanisms and the prepayment method for healthcare financial contributions as a means for increasing population coverage (36). Raising new taxes and improving the tax administration strategies can help increase the fiscal space for health (63). Moreover, governments must ensure appropriate and fair distribution of healthcare systems of good quality for the population and maintain sustainable funds of health programs or activities (36). External funding can be another important source of public funding for health particularly in lower middle-income countries and upper middle-income countries that are hosting huge numbers of refugees, but efforts are needed to ensure their predictability (11, 64).

To meet the increased demands for healthcare services and the needed development in the delivery of certain types of services, countries of the MENA region must develop clear policies and strategies to strengthen the role of the private health care sector (13). The governments of these countries must thoughtfully use the private sector facilities and fully regulate them to serve the population health needs and to complement the overstretched public health sectors (3, 4). Indeed, the private sector is expected to play a key role in providing health care in both high- and low-income courtiers in the region, as well as in conflict and post conflict settings as it can provide health care for refugees (3, 13, 16).

Reconfiguring the MENA health systems is needed to effectively integrate the provision of promotional and preventative services with curative and support services (4). This would require development and adoption of suitable policies and good management of human resources (4). Efforts are also needed to mitigate the risk factors underlying the development of NCDs to limit the disease burden and direct and indirect healthcare costs (4, 13). There must be a focus on developing innovative paradigms and methods to re-organize the health care delivery system (4). This can include good collaboration between different providers and stakeholders, maximizing skills mix and improving the training of health professionals, effective use of primary and acute care services, and rationale use of available medical and pharmaceutical technologies and the adequate integration of new ones (4). Evidence has shown that the investment in the delivery and quality of basic health services such as preventative and primary care can result in improvements in health outcomes and equitable access to care (22). Therefore, shifting investment to ensuring basic, primary care can assist low-income countries, as well as sub-national territories that are challenged by resources constraints (22). On the other hand, it would be beneficial for middle-income countries that apply a combination of governmental subsidies and social health insurance such as Egypt, Tunisia and Morocco to ensure the effective functioning of the targeting mechanisms and availability of awareness programs for eligible beneficiaries (22). Middle to upper middle-income countries which implement social health insurance schemes are advised to continue fine-tuning systems in place to address institutional-level issues and optimize the outcomes (22).

Development of new model of care such as decentralized and flexible healthcare services, and providing support for patients to move from provider-centric care to patient-, primary-, and community-centric care are among the strategies that can help overcome issues such as the increased aging population and the disproportionate concentrations of resources between urban and rural areas (10). Integration of healthcare services such as formation of multidisciplinary team and developing initiatives that aim for a greater involvement of the community can be a key step to coordinate health care delivery and decrease the burden on the health systems (10). Another important element that must be considered involves encouraging patient-based care and developing approaches to the education and empowerment of patients to help them play an active role in their own care (4, 10).

Health staff development has been fundamental to the improvement of quality of health care (10). A well-trained health workforce is also needed to strengthen the public health infrastructures in the MENA region (5). This would require development of health school curricula to link health workforce education and training with the health needs of the population (4, 5). Health systems must ensure that the health practitioners are kept up to date with medical advances and technological developments (10). This would require careful consideration to workforce education and training, recruitment, professional development and establishment of leadership roles (10).

Addressing the challenges facing MENA health systems will require creating comprehensive health information systems to provide accessible information for planning, managing and implementing services (4). Health IT solutions are essential for modern health systems and can be useful assists for health system reform (39, 40). Adopting e-health technology has been improving patient-centered care by enhancing electronic data storage, management and capacity, while providing readily available information to clinicians, patients and healthcare providers (10).

To overcome health-system related challenges, resources, political commitment and management capacity must be invested to support the available public health measures and functions or to create new ones if they are absent (4). Perhaps the most crucial of these functions would be public information and education, intersectoral policy making, and quality assurance and improvement, which require transparent governance structures and good coordination between the various entities beyond the health sector (4). Benchmarking of the health systems within the region can be a useful tool to guide the internal health reform of the individual countries (15). It can identify the objectives that must be accomplished in terms of the health sector development and the areas that the reform process must address to overcome low performance in health outcomes (15).

Braithwaite et al. (18, 19) outlined the common factors that are linked to the success in their overview of 60 country case studies on health reforms. These included starting a small scale initiatives that can result in system-wide reforms, supporting the role of data and IT in the delivery of efficient and appropriate care, stakeholders concrete action and collaboration to mediate the reform and situating the patients at the center of reform process (18, 19). Starting small and modest scale projects and piloting or testing the reform initiatives can prepare the setting to implement larger scale reforms over time (18, 19). Attention must be directed toward the integrated use of IT, operational data storage and transmission, and existence of accessible decision support tools and databases to enable communication and information exchange among decision makers and stakeholders in a timely, effective way (18, 19). System improvements can be mediated through coordinating collaborative relationships among multiple stakeholders, applying evidence-based decisions and implementing clear principles of reform design (18, 19). Centering the patients, their wellbeing, needs and experiences at the heart of the reform process is perhaps the most vital factor to ensure the success of the reform enterprise (18, 19).

The future health systems are shaped by universal trends (10, 65). These include the genomics revolution that will result in development of new system of care, evolution of emerging technologies such as e-health capacities, global demographical dynamics due to increasing and mobilization of population which constantly alter demands for health services, and the development of new model of care in response to issues such as increased aging population (10). This would necessitate the development of sustainable health systems that can overcome the evolving challenges and respond to the stressors resulting from accelerated changes (10). Countries in the MENA region are advised to develop their health systems to keep pace with these universal trends.

## Conclusion

The MENA region is among the highly dynamic areas in the world, full of both opportunities and challenges (16). The evidence describing health system reforms in the MENA region has been scarce and underrepresented in the literature. This review article attempts to add to the existing published literature and to provide examples on improvements made on the performance of the health systems in the MENA countries. It also highlights the plethora of challenges facing the health systems of the region. As challenges continue to advance, the methods of health reform will continually evolve to overcome these challenges (19). Accordingly, health systems of the MENA region must develop their adaptability and responsiveness to meet the increasing demands for health care by the population while overcoming the financial shortages and increasing challenges.

## Author contributions

MK reviewed the literature to collect the relevant evidence, evaluated the evidence, organized the findings and wrote the first draft of the review article. MJ, AC, and LG contributed to the development of the outline of the review article and evaluation of the collected evidence, revised the manuscript and provided suggestions for expansion and improvement. All authors read and approved the final version submitted for publication.
